# Dosimetric impact of the 160 MLC on head and neck IMRT treatments

**DOI:** 10.1120/jacmp.v15i6.4770

**Published:** 2014-11-08

**Authors:** Prema Rassiah‐Szegedi, Martin Szegedi, Vikren Sarkar, Seth Streitmatter, Y. Jessica Huang, Hui Zhao, Bill Salter

**Affiliations:** ^1^ Department of Radiation Oncology University of Utah Salt Lake City UT USA

**Keywords:** 160 MLC, 120 MLC, head and neck treatments, IMRT, MLC leaf width

## Abstract

The purpose of this work is to investigate if the change in plan quality with the finer leaf resolution and lower leakage of the 160 MLC would be dosimetrically significant for head and neck intensity‐modulated radiation therapy (IMRT) treatment plans. The 160 MLC consisting of 80 leaves of 0.5 cm on each bank, a leaf span of 20 cm, and leakage of less than 0.37% without additional backup jaws was compared against the 120 Millennium MLC with 60 leaves of 0.5 and 1.0 cm, a leaf span of 14.5 cm, and leakage of 2.0%. CT image sets of 16 patients previously treated for stage III and IV head and neck carcinomas were replanned on Prowess 5.0 and Eclipse 11.0 using the 160 MLC and the 120 MLC. IMRT constraints for both sets of 6 MV plans were identical and based on RTOG 0522. Dose‐volume histograms (DVHs), minimum dose, mean dose, maximum dose, and dose to 1 cc to the organ at risks (OAR) and the planning target volume, as recommended by QUANTEC 2010, were compared. Both collimators were able to achieve the target dose to the PTVs. The dose to the organs at risk (brainstem, spinal cord, parotids, and larynx) were 1%–12% (i.e., 0.5–8 Gy for a 70 Gy prescription) lower with the 160 MLC compared to the 120 MLC, depending on the proximity of the organ to the target. The large field HN plans generated with the 160 MLC were dosimetrically advantageous for critical structures, especially those located further away from the central axis, without compromising the target volume.

PACS number: 87.55 D‐

## I. INTRODUCTION

The use of intensity‐modulated radiation therapy (IMRT) in the treatment of head and neck squamous cell carcinoma (HNSCC) is prevalent in today's radiation oncology practice. The highly conformal doses achieved with IMRT plan results in greater tissue sparing and toxicity with similar or better locoregional control when compared with three‐dimensional conformal treatments.^1^, ^2^

The multileaf collimator is a common tool which is used to modulate these IMRT treatment beams, and the plan quality and delivery of treatments are dependent on the dosimetric and mechanical properties of the MLC. Dosimetric properties such as leakage, penumbra, tongue and groove, and leaf resolution directly affect plan quality, whereas the leaf span and MLC leaf speed affect efficiency and duration of treatments.

One of the most common commercially available and used MLCs for IMRT is the Varian 120 leaf Millennium. The Varian 120 MLC has a 5 mm leaf resolution at isocenter for the central 40 leaves (i.e., central 20 cm), and 10 mm for the remaining outer leaves. The more recently released Siemens 160‐leaf MLC has a leaf resolution of 5 mm at isocenter for the entire field. Besides the finer leaf resolution,^(3)^ a much lower overall leaf leakage and transmission are also attributed to this MLC. The advantage of this finer resolution MLC for the entire field size can only be manifested if the IMRT field utilizes these leaves. A common site where large field IMRT is typically employed and these outer leaves are used for modulation is head and neck.

HNSCC IMRT plans have comparatively large field sizes with various small organs at risks (OARs) (e.g., parotid, larynx, brainstem, spinal cord) spread over the range of the treatment field. Since the treatment of these patients generally involves the entire cervical lymphatic drainage, as well as the gross tumor volumes, the field size used is also large, up to 25 cm in length. Depending on where the isocenter is placed, a significant part of the field can be shaped by the 10 mm leaves if the 120 MLC is used. The question then arises as to whether better target coverage and increased critical structure shielding can be achieved if smaller leaves are used for the entire treatment field. To the best of our knowledge, there have been no published studies to date comparing these two MLCs for large‐field IMRT treatments.

The purpose of this work is to characterize potential changes in plan quality when employing the finer leaf resolution and lower leakage of the new Siemens 160 MLC, and to determine if these changes are dosimetrically significant for HNSCC IMRT treatment plans.

## II. MATERIALS AND METHODS

### A. Brief description of MLCs

The Varian Millennium MLC (Varian Medical Systems, Palo Alto, CA), mounted on a Varian 23EX linear accelerator at our center, consists of 60 leaf pairs which are carriage mounted, has a 15 cm leaf travel, and allows interdigitization. In combination with carriage motion, the MLC is positioned under a set of conventional X and Y jaws with a patient clearance of 334 mm (i.e., distance from lower edge of collimator to isocenter is 334 mm). The outer 10 leaves on each side of a leaf bank project a 10 mm thickness, while the remaining inner 40 leafs are 5 mm thick at isocenter. The MLC leaves are designed with a tongue‐and‐groove (0.4 mm groove) and 5.5 cm height, resulting in an average leakage and transmission of 2%.^4^, ^5^

The single focused 160 MLC in our center is mounted on an ARTISTE linear accelerator (Siemens Medical Solutions, Malvern, PA). Each leaf projects a 5 mm thickness at isocenter, and allows for 20 cm leaf travel and interdigitization. In combination with carriage motion, a leaf can be positioned anywhere in the maximum field size of 40cm×40cm. The MLC works with a set of Y jaws and the X jaws are replaced by the 80 leaf pairs. This results in a short source to lower edge of collimator distance of 460 mm, thus enabling a 430 mm patient clearance. The tilted leaves design and the 9.5 cm leaf height results in average radiation leakage and transmission of less than 0.37%^(3)^ without additional back up jaws (see Table 1).

**Table 1:** A comparative list of some of the characteristics of the two MLCs. Both the 160 MLC and the 120 MLC are in clinical use in our department.

	*160 MLC on ARTISTE*	*120 MLC on 2300 EX*
Source collimator distance	460 mm	535 mm
Patient clearance	430 mm	334 mm
Leaf resolution	5 mm leaf entire 40 cm field	5 mm central 40 leaves and 10 mm remaining outer leaf
Leaf End	S‐shaped	C‐shaped
Leaf height	95 mm	55 mm
Penumbra (80 ‐ 20%)	3.6 mm	3.2 mm
Leakage and transmission	0.37%	2.00%
Inter‐digitization	Yes	Yes
Method to avoid a direct ray in between leaves	Tilted alternating pattern of upper and lower leaf	tongue and groove
Max Leaf Speed	$40\;{\text{mms}}^{-1}$	$20\;{\text{mms}}^{-1}$
Leaf Span	200 mm	145 mm
Step and shoot IMRT	Yes	Yes
Dynamic IMRT	No	Yes

It should be noted that this study compares the 120 MLC on a Varian 2300 Clinac and the 160 MLC on the Siemens ARTISTE system. Although the underlying 6 MV beam energy is comparable between the two systems, differences in terms of beam profiles are inherent in this comparison.

### B. Patient study

CT image sets of 16 patients, previously treated with stage III and IV HNSCC, were replanned using the Prowess Panther treatment planning system (version 5.0; Prowess Inc., Chico, CA) using the 160 MLC on an ARTISTE Siemens linear accelerator and the 120 MLC (Varian Medical Systems) on a 2300EX Varian accelerator. All 16 patients were also replanned on Eclipse (version 11.0; Varian Medical Systems) to preclude vendor bias in the conclusion on this study.

The primary tumor and involved nodes (PTV1), subclinical disease sites (PTV2), spinal cord, brainstem, larynx, and parotids were contoured. Isocenter is typically placed at the level of C2/C3. Identical IMRT constraints based on the guidelines outlined in RTOG 0522 were used for the brainstem, spinal cord, larynx, and parotids during the optimization for plans. If there was an overlap of the target and critical structure, target coverage was given priority. All plans were normalized such that 95% of the volume of PTV1 receives the prescription dose of 70 Gy and PTV2 receives at least 93% of the prescribed dose. Five to seven beams (6 MV) were used to generate plans with field sizes ranging from 16 to 20 cm in width and 16 to 24 cm in length. Identical beam configurations were used for IMRT plans created for both collimators. All plans were planned using the step‐and‐shoot technique. The plans were planned by a single physicist for both the planning systems for both the collimators. The planner had vendor training and has similar experience planning on both the planning systems.

The dose‐volume histograms (DVHs) and mean and maximum dose to the planning target volumes (PTV) were compared. Dose indices used for organ at risk comparison (OAR) were based on recommendations from QUANTEC 2010. The dosimetric parameters that are compared for the brainstem are maximum dose ($D_{\text{max}}$), dose to $1\;{\text{cm}}^{3}$ ($D_{1\text{cm}3}$) and mean dose ($D_{\text{mean}}$).^(6)^ $D_{\text{max}}$ and $D_{1\text{cm}3}$ were compared for the spinal cord.^(7)^ Mean doses are compared for both larynx^(8)^ and parotids.^(9)^ A paired *t*‐test (95% confidence interval) was used to indicate if the difference in dose created by both these plan is statistically significant.

### C. Planning systems

Prowess 5.0 uses the direct aperture optimization (DAO) method to produce IMRT plans, which considers the physical geometry and energy properties of the machine; hence, the need for leaf sequencing is eliminated. The typical number of segments per beam used for planning the above studies in Prowess is approximately 15. Eclipse 11.0 uses the dose volume optimizer (DVO), which employs a simple gradient optimization with line minimization. A leaf motion calculator, which is called Multiple Static Segments (MSS), is then used to calculate the leaf pattern. The typical number of segments generated per beam used to produce the final dose distribution is approximately 20 for this study. A dose grid size of 2.5 mm was used for dose calculation for both the planning systems

Both the 120 MLC on the Varian 23EX and the 160 MLC on the ARTISTE are fully commissioned on Eclipse 11.0 and are being used clinically. A 2%/mm agreement criterion between measured and modeled beam data was used to commission the systems. A minimum of 90% pass rate on absolute gamma index is our in‐house criterion for all IMRT plans regardless of the device used for validation.

On Prowess, the 160 MLC on the ARTISTE is fully commissioned with the same criteria as the Eclipse system. The beam profiles and depth doses of the 120 MLC on the 23EX meet the agreement criteria of 2%/mm between the measured and modeled beam data. However, no clinical plans have been generated on the 23EX using Prowess. In order to gauge the accuracy of the beam model of the 120 MLC on the 23EX, two head and neck IMRT plans were generated on Prowess for both the 120 MLC on the Varian 23EX and the 160 MLC on the ARTISTE. Measurements were made with solid water, EDR film, and an ion chamber (PTW 31010, 0.125 cc chamber; PTW, Freiburg, Germany).

## III. RESULTS

IMRT plans generated on Prowess using the 120 MLC on the 23EX seem to validate well. Table 2 shows the agreement between measured and calculated dose for two head and neck IMRT plans.

**Table 2:** The agreement between measured and calculated dose for two IMRT plans with the Prowess planning system.

		*Prowess*	
		*120 MLC*	*160 MLC*
Plan#1	Ion chamber deviation / % (measured ‐ calculated)	1.4	−1.6
	Gamma index (3%/mm)	92.5	92.7
Plan#2	Ion chamber deviation / % (measured ‐ calculated)	−1.4	1.1
	Gamma index (3%/mm)	94.5	93.3

The mean PTV1 size was $431.9\;{\text{cm}}^{3}$ ($109.6–982.9\;{\text{cm}}^{3}$) and the mean PTV2 size was $163.5\;{\text{cm}}^{3}$ ($9.8–442.1\;{\text{cm}}^{3}$). For both collimators on both planning systems, 95% of PTV1 received 66.5 Gy and 93% of PTV2 received at least 65.1 Gy. The mean doses to PTV1 were 71.3 Gy and 71.7 Gy with the 160 MLC and 120 MLC, respectively, with the Prowess planning system. With the Eclipse planning system, the mean doses to PTV1 were 70.4 Gy and 70.8 Gy with the 160 MLC and 120 MLC, respectively. The differences in the minimum, maximum, and mean dose for the PTV for 160 MLC and the 120 MLC were statistically insignificant for both the planning systems.

For the 16 patients compared here, the average maximum dose, mean dose, and $D_{1\text{cm}3}$ to the brainstem were lower by 2.3 Gy, 1 Gy, and 1.6 Gy, respectively, when using the 160 MLC on both the planning systems. The results are tabulated in Table 3. All of these differences were found to be statistically significant.

**Table 3:** Difference (dose 120 MLC ‐ dose 160 MLC) maximum, mean, and $D_{1\text{cm}3}$ dose in brainstem between the two collimators.

	*Eclipse 11.0*			*Prowess 5.0*		
*BrainStem*	*Max. Dose*	*Mean Dose*	$D_{1\text{cm}3}$	*Max. Dose*	*Mean Dose*	$D_{1\text{cm}3}$
Average Difference/Gy (Range)	2.0	0.6	1.4	2.6	1.3	1.8
	(−0.6–3.8)	(−1.1–5.2)	(−1.4–4.3)	(−1.6–4.4)	(0.2 ‐3.8)	(0.1–4.4)
p‐value	0.04	0.03	0.04	0.04	0.01	0.02

On average, the maximum dose to the spinal cord was 1.5 Gy (up to 8.3 Gy) lower for 160 MLC, whereas the $D_{1\text{cm}3}$ was on average 1.5 Gy (up to 6.6 Gy) lower for the 160 MLC. The differences in maximum dose and $D_{1\text{cm}3}$ were found to be statistically significant as indicated by p‐values shown in Table 4.

**Table 4:** Difference (dose 120 MLC ‐ dose 160 MLC) in maximum and $D_{1\text{cm}3}$ for spinal cord dose between the two collimators.

	*Eclipse 11.0*		*Prowess 5.0*	
*Spinal Cord*	*Max. Dose*	$D_{1\text{cm}3}$	*Max. Dose*	$D_{1\text{cm}3}$
Average Difference/Gy (Range)	1.6	1.7	1.5	1.2
	(0.0–8.3)	(0.5– 6.6)	(0.5–5.6)	(0.5–5.5)
p‐value	0.01	0.01	0.04	0.02

On average, the mean larynx dose for the 160 MLC was 1.8 Gy lower compared to the 120 MLC, a statistically significant difference. On average, the mean right parotid and left parotid dose for the 160 MLC was 1.2 Gy and 1.4 Gy lower, respectively, compared to the 120 MLC, with a p‐value of 0.01 for both the right and left parotid, as shown in Table 5.

**Table 5:** Difference (dose 120 MLC ‐ dose 160 MLC) in mean dose in the left parotid, right parotid, and larynx between the two collimators.

	*Eclipse 11.0*			*Prowess 5.0*		
*Mean Dose /Gy*	*Lt. Parotid*	*Rt. Parotid*	*Larynx*	*Lt. Parotid*	*Rt. Parotid*	*Larynx*
Average Difference/Gy (Range)	0.7	1.1	1.7	1.7	1.3	1.8
	(0.4–3.1)	(0.2–3.9)	(0.4–4.1)	(0.7–6.9)	(0.5–5.0)	(0.2–5.2)
p‐value	0.01	0.1	0.01	0.01	0.01	0.02

**Figure 1:**
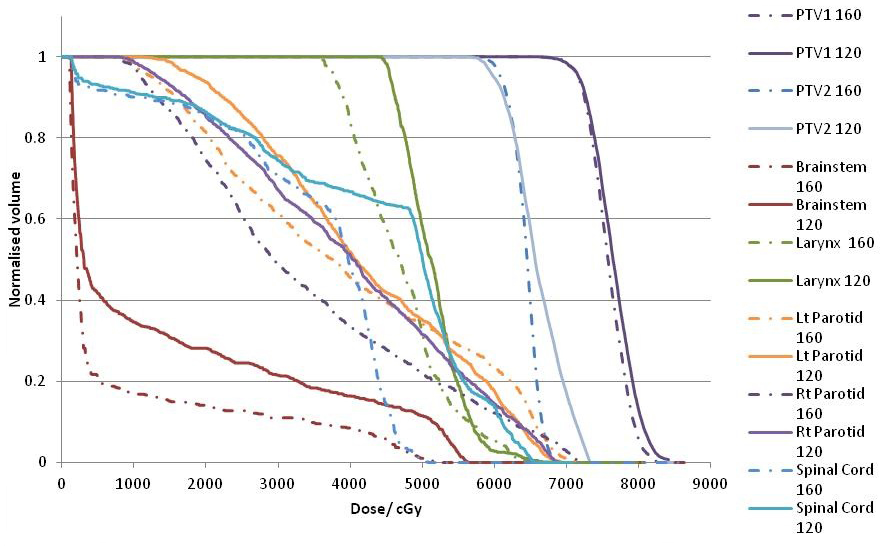
Typical DVH for plans calculated with the 160 MLC and 120 MLC.

**Figure 2:**
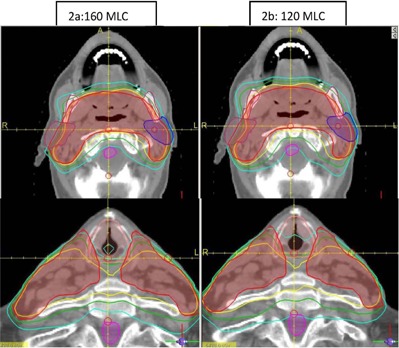
The left (a) and right (b) panels show the comparative dose distribution for the 160 MLC and 120 MLC, respectively. Note the dose sparing on the larynx and spinal cord with the 160 MLC.

**Figure 3:**
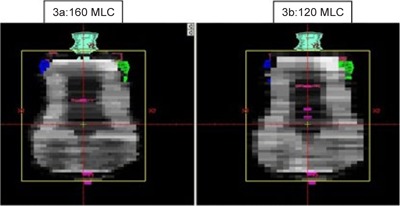
A typical fluence map for the 160 MLC (a) and 120 MLC (b) for the same patient at 180° beam angle.

Figure 1 shows a typical DVH with both plans required to produce similar target coverage. A superior and inferior axial image slice depicting spinal cord and larynx sparing with the 160 MLC is shown in Fig. 2. The difference in fluence maps due to different MLC resolution 10 cm superior to the central axis is clearly visible in Fig. 3. The PTV conformity between the 160 MLC and the 120 MLC were similar as the isocenter was placed close to the center of the PTV where both MLCs have the same leaf resolution of 0.5 cm. Besides dosimetry, the plans generated on the 120 MLC had multiple split fields resulting in almost double the number of treatment fields, potentially increasing delivery time.

## IV. DISCUSSION & CONCLUSIONS

On average, the critical structure (brainstem, spinal cord, larynx, and parotids) doses were lower with the 160 MLC compared to the 120 MLC, and this difference is shown to be statistically significant for both planning systems. This lower dose is probably due to the finer resolution and the lower MLC transmission of the 160 MLC. In head and neck treatments, small critical structures are in very close proximity to, or sometimes even in contact with, the target, where the resolution of the MLC at the interface of the target and critical structure plays a role in the optimization of target coverage and critical structure shielding. For example, a 1 cm leaf at the boundary of the target and critical structure (i.e., leaf over both the target and critical structure) has to be opened in order to provide target coverage, but that exposes both the target and critical structure. However, if 0.5 cm leaves are used, one will have the option to open one leaf and close the other to ensure that the critical structure is shielded whereas the target is exposed. This effect is clearly seen in the above results in the maximum and $D_{1\text{cm}3}$ doses for in the brainstem and spinal cord which generally occurs at this boundary area, where the 160 MLC seems to be better at shielding the critical structure without compromising on target coverage. The other reason for the lower dose mainly in the mean dose is the lower transmission of approximately 0.37% for the 160 MLC, compared to 2.0% for the 120 MLC.

In terms of target (PTV1 and PTV2) coverage, the dose distribution of both the MLCs are similar, as expected, since a significant amount of the target is covered with the same leaf width on both the collimators. This is due to the fact that isocenter is placed at the level of C2/C3 and only the superior 2 to 3 cm of the target is covered by different leaf widths (i.e., 0.5 cm on the 160 and 1 cm on the 120 MLC). Zwicker et al.^(10)^ compared the 160 MLC and the 80 leaf MLC (1 cm leaf width over the whole range) for head and neck targets, and they reported improved target coverage with the 160 MLC. They also reported reduced dose to the spinal cord, brainstem, and parotid glands, which is consistent with our findings. Yoganathan et al.,^(11)^ however, compared the Varian 120 leaf MLC and the Varian 80 leaf MLC for five head and neck patients and reported no appreciable difference in terms of target coverage or OARs (parotids) sparing.

The magnitude of difference in dose for the OARs between the 160 MLC and 120 MLC is similar for both planning systems, as seen in Tables 3, 4, 5. Eclipse 11.0 does not interdigitize the MLC leaf for the 160 MLC, although the 160 MLC is capable of interdigitization. Although, interdigitization is done only for the 120 MLC on Eclipse, the difference in plan quality is similar as the average total number of segments between the 160 and 120 MLC are similar. The difference in being able to interdigitize may show up in the efficiency of plan delivery.

The large field head and neck plans generated with the 160 MLC are dosimetrically advantageous for critical structures, especially when they are located further away from the central axis in the inferior–superior direction, without compromising the target volume coverage. Besides, the 160 leaf MLC leaf travel of 20 cm also reduces the number of split fields, which can reduce delivery time.
